# Case Report: Huge Dumbbell-Shaped Primary Hydatid Cyst Across the Intervertebral Foramen

**DOI:** 10.3389/fneur.2020.592316

**Published:** 2020-11-24

**Authors:** Yu Tian, Mei Jiang, Xin Shi, Yujun Hao, Lei Jiang

**Affiliations:** ^1^Neurosurgery Department, The First Affiliated Hospital of Xinjiang Medical University, Urumqi, China; ^2^Medical Imaging Center, Huazhong University of Science and Technology Union Shenzhen Hospital, Shenzhen, China

**Keywords:** hydatid cyst, echinococcosis, spinal canal, intervertebral foramen, case report

## Abstract

Primary hydatid cyst of the spinal canal is extremely rare. We reported a 42-year-old Kazakh man with right lower back pain and weakness in both lower limbs for 2 months, who lived in the pastoral area. Clinical examination revealed that the patient had no cysts on other organs and no previous medical history except for a huge cyst inside and next to the vertebrae. MRI examination revealed a huge dumbbell-shaped primary cyst across the intervertebral foramen. Pathological examination after operation confirmed a fine-grained hydatid cyst disease. Therefore, in the pastoral area, doctors should be alert to the occurrence of hydatid cyst disease if patients complained about progressive back pain and lower limb weakness or other spinal cord compression symptoms. Once hydatid cysts in other organs or systems were detected, the occurrence of the disease could be highly suspected. Complete resection is an effective treatment for hydatid cyst disease.

## Introduction

Hydatid cyst disease, also known as echinococcosis, is a parasitic disease that affects humans and other mammals such as sheep, dogs, mice, and horses, relatively common in livestock areas. Humans may become infected by direct contact with the definitive host (dogs) or by ingestion of food infected with the parasite's eggs (1). In clinical practice, the liver was the most susceptible organs of echinococcosis infection, accounting for about 75% of cases; the lung follows, accounting for about 15%; other organs such as the brain can also be affected, but <10%. And only 0.5–2% of patients have bone involvement, with half of them mainly affecting the spine (1, 2). The most common affected sites of the spine were the lumbar, thoracic, sacral, and cervical vertebrae, which were mainly affected by the vertebral body (3). And echinococcosis originating from the spinal cord or spinal canal was extremely rare. Spinal hydatid cyst disease usually presents as cauda equina symptoms or symptoms of spinal cord compression. And it is typed into five categories: (1) primary intramedullary hydatid cyst; (2) intradural extramedullary hydatid cyst; (3) extradural intraspinal hydatid cyst; (4) hydatid disease of the vertebrae; and (5) paravertebral hydatid disease (4, 5). To the best of our knowledge, primary hydatid cyst of the spinal canal across the intervertebral foramen has been rarely and seldom reported in the literature. This case report describes a rare presentation of primary extradural intraspinal and paravertebral connected primary hydatid disease. It was the first time that the initial diagnosis of the patient was the hydatid cyst disease, but no hydatid involvement was found in other organs except the spinal area, the serological test (ELISA) of echinococcosis was also negative. The purpose of this case report is to share our experience in the diagnosis and treatment of hydatid cyst disease and to review the relevant literature.

## Case Presentation

### Presentation and Examination

A 42-year-old Kazakh patient was admitted to the hospital with pain in his right lower back and weakness in both lower limbs with no inducement for 2 months. The pain was aggravated after activity and was relieved after rest, the Visual Analog Scale (VAS) pain score was 7. Neurological examination revealed bilateral lower extremity weakness: left muscle strength was 3/5, the right was 4/5. With brisk tendon reflexes and negative Babinski sign, the remainder of the physical examination was unremarkable. Besides, the patient grew up in a pastoral area with close contact with cattle, sheep, horses, and other livestock.

### Neuroimage

**Figure 1:**
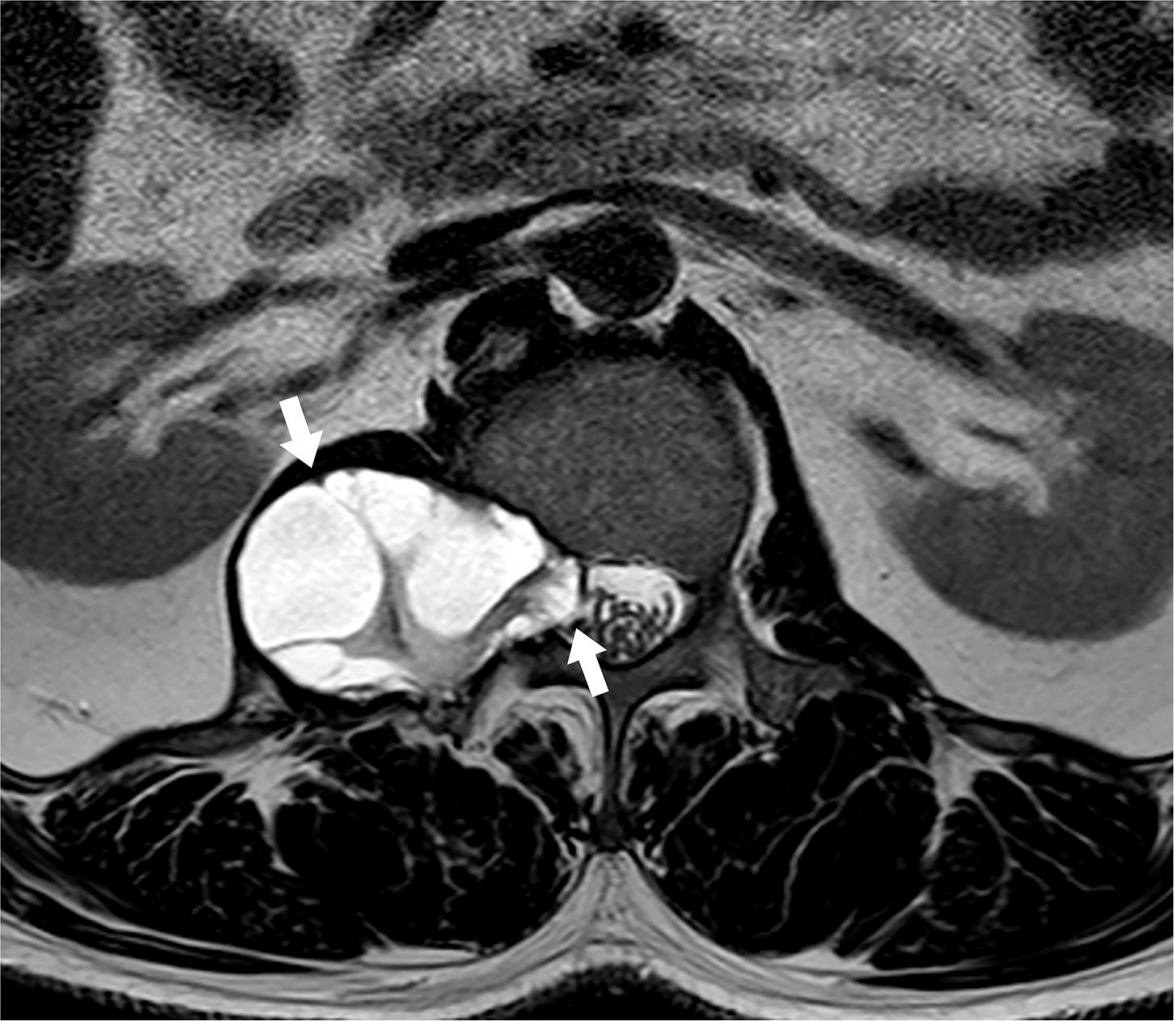
MRI T2WI (axis image) showing hyperintense signal of an oval solid-cystic cavity at the right side of the vertebral body and across through the enlarged L1~L2 intervertebral foramen. Left arrow: The part outside of the spinal canal. Right arrow: The part passing through the intervertebral foramen and inside of the spinal canal.

**Figure 2:**
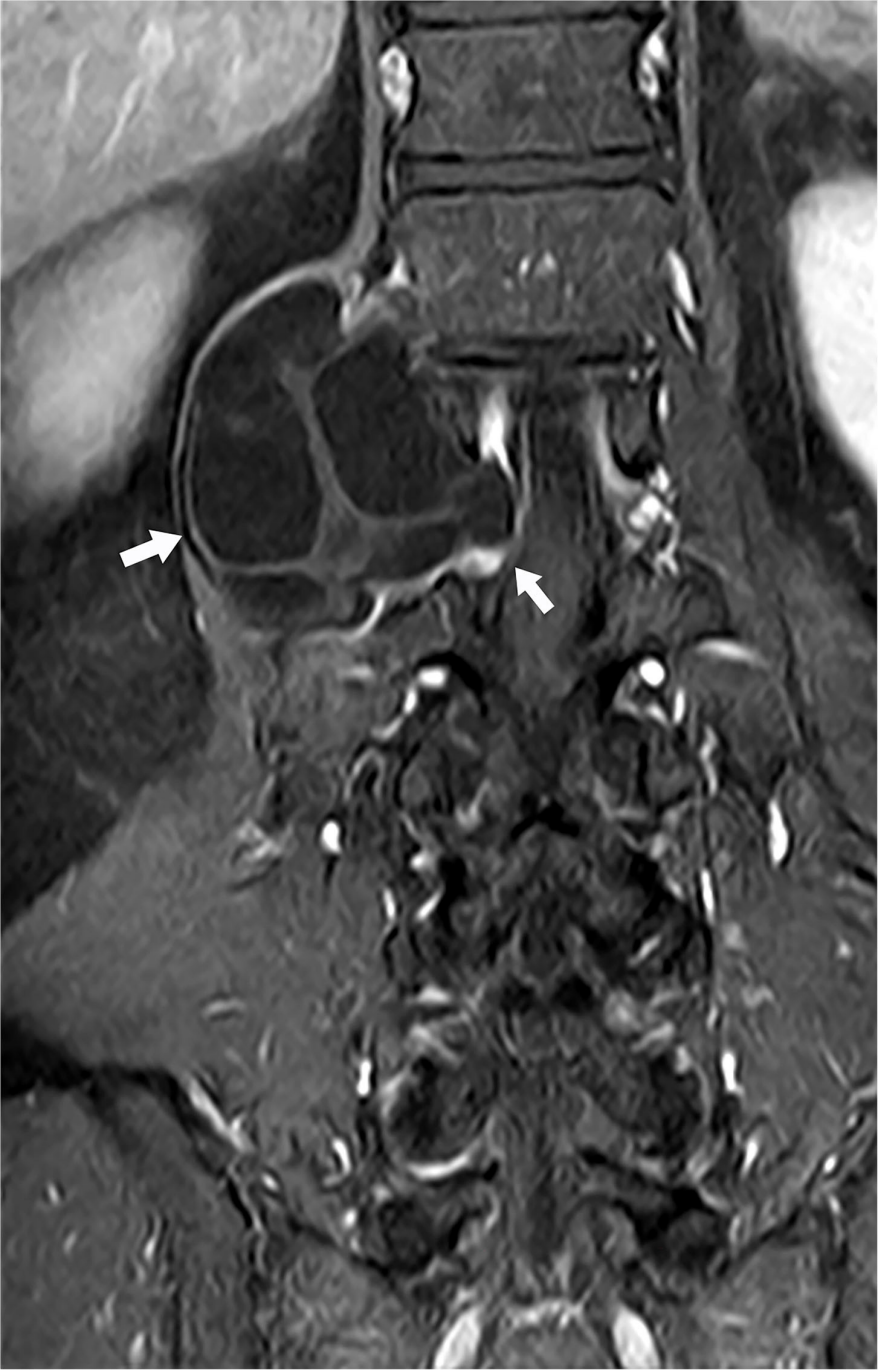
MRI T1WI (coronal image) showing corresponding intervertebral foramina around the cavity was enlarged. Left arrow: The part outside of the spinal canal. Right arrow: The part passing through the intervertebral foramen and inside of the spinal canal.

The CT scan showed the L1 vertebral body and peripheral bone were hyperostotic (smooth edge), the relevant intervertebral were also enlarged. The MRI scan performed an oval solid-cystic cavity at the right side of the vertebral body of the T12~L2 vertebrae and the enlarged L1~L2 intervertebral foramen, the cystic cavity was hypointense on T1-weighted-image (T1WI), hyperintense on T2-weighted-image (T2WI), and T2-fat-suppression-image (T2FS). The multiloculated cystic lesions could be seen inside and had clear boundaries with surrounding tissues. Corresponding intervertebral foramina around the cavity was enlarged, which compressed the conus medullaris of L1 and extended into the adjacent psoas (Figures 1, 2). The L4~L5 and L5~S1 intervertebral disc protruded to the left rear. The enhancement MRI image showed the abnormal signals and significantly enhanced septum on the right side of the T12~L2 paravertebral and the right side of the L1~L2 intervertebral foramen. According to MRI and pastoral area life history, we highly suspected it as the hydatid cyst disease. The CT and B ultrasound examination of other body parts showed no signs of systemic cyst.

### Operation

We used the posterior straight incision approach of T12~L2. The incision was made to expose the T12~L2. The laminectomy was performed for the right part of the vertebral plate and inferior articular process of the T12, the right vertebral plate and attachment of the L1, and superior articular process at the right side of the L2. Entering the incision, the oval solid-cystic cavity with clear boundaries located at extradural (across the intervertebral foramen and the dumbbell extension was intraspinal) and had compressed the spinal cord to the left ventral side. The lesion has high tension and tight adhesion to the dura mater and intervertebral foramina. Gauzes were used to protect the incision and the surrounding tissues, we carefully cut the ectocyst to decompress, a whitish, pearly, translucent cystic endocyst was found. After careful separation and loosening, we scratched the edge of the cyst to remove the endocyst as a whole carefully. And loosen the attachment of the dural sac, ensure that it was intact and pulsates well. Then used hydrogen peroxide and 5% hypertonic saline to inject for 10 min, checked again whether there was residual endocyst. Peeled off most of the ectocyst carefully, followed by hydrogen peroxide, 5% hypertonic saline, and dexamethasone irrigated the operative site again to avoid relapse and anaphylactic reaction. Finally, spinal fusion and fixation of T12~L2 have been performed and the wound was closed in layers.

### Histopathological Examination

**Figure 3:**
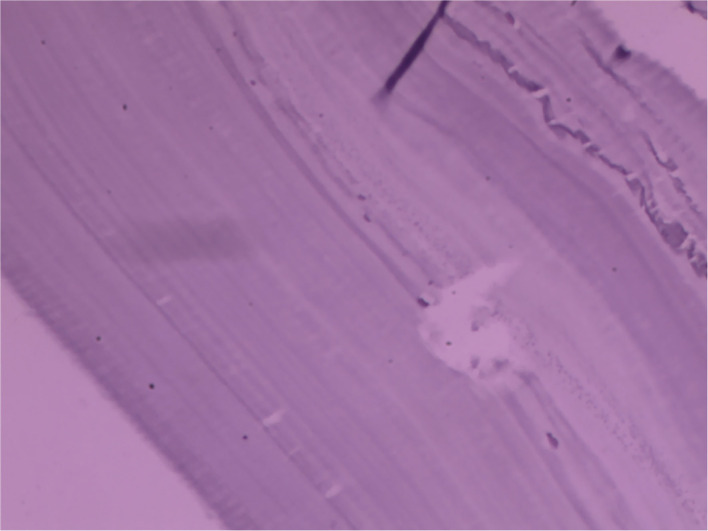
In the histopathological examination, after Hematoxylin and Eosin (H&E) staining of the cyst, endocyst's germinal layer could be seen.

In histopathological examination, we found that after Hematoxylin and Eosin (H&E) staining of the tissue, granulomas and inflammatory granulation tissue could be seen, consisting of proliferative fibers, vascular epithelioid cells, and multinucleated giant cells. The hydatid and endocyst's germinal layer could be found (Figure 3). Chronic interstitial cells are present in the stroma and support a severe inflammatory reaction. All histopathology features above revealed it was the hydatid cyst disease.

### Post-operative Course

The patient recovered well after surgery, the muscle strength of both lower limbs was restored to level 5/5, and the pain was almost completely relieved (VAS score from 7 to 2), Albendazole session was continued for 12 months (10 mg/kg body weight/day) as for standard anti-infectious therapy of Echinococcus. During the follow-up and reexamination for 6 months after surgery, the patients did not show the same similar symptoms, and there was no sign of recurrence on imaging evaluation. We advise the patient to take the medication on time and to review for further imaging every 6 months.

## Discussion

Spinal hydatid disease is rare and was rarely reported in the literature. The main infectious source of this disease is livestock, most of which are caused by the invasion of Echinococcosis Granulosa into the human body, and a few are caused by Echinococcosis Multilocularis. The former is the more benign form and is characterized by cyst formation, whereas the latter is not encapsulated and presents as a ramifying, porous, and necrotic mass. The prognosis for the multilocular form is extremely poor, with an almost 100% mortality rate (6). Spinal hydatid disease is mostly hematogenous metastasis, due to the rich blood circulation and the slowly flowing blood of the spine area. Therefore, spinal involvement is more common in bone hydatid disease, accounting for about 50% (7). Spinal hydatid disease can occur in any segment of the spine, while 50% of the vertebral involvements are seen in the thoracic area and 20% are in the lumbar area, sacral and pelvic involvement are rare (3). It can be originated in the vertebral canal wall, or it can invade into the spinal canal by sacrospinous muscle hydatid through the intervertebral foramen and compress the spinal cord. Due to the limitation of the surrounding bone wall, the development is slow, but the proliferation and spread can be outward along with the Haversian osteon lamella system. When the cyst breaks through the cortical bone into the spinal canal, the compression of the spinal cord or nerve root may occur, due to various shapes of the space formed by the spinal canal and its surrounding soft tissues, the different shapes of the hydatid cyst can be formed.

In this case, vertebral canal involvement was primary, and no signs of hydatid were found in the vertebrae and other organ systems, the serological test (ELISA) was negative, which made the case extremely rare. In our literature review, Parvaresh et al. (8) reported a dumbbell hydatid cyst of the spine, but in their case, spinal involvement of hydatid disease was considered secondary, because the primary lesion was located in the liver, whereas in the presented case spinal involvement was primary. Karakasli et al. (6) reported a primary dumbbell hydatid cyst of the thoracic spine, but the part of the paravertebral was smaller than that in our case. For the case we reviewed, primary hydatid infestation of the spine without any other systemic involvement can be explained through the direct Porto-vertebral venous shunt theory: in rare instances, the disease begins from the extradural area, suggesting that the parasite's embryo is possibly being carried through the Porto-vertebral venous shunts (9–11). The disease has been reported for various names in different literature: including hydatid disease invasion of the spinal canal, extradural intraspinal hydatid cyst, spinal hydatid disease or hydatid cyst, etc., currently collectively known as spinal hydatid disease.

The early diagnosis of this case for primary hydatid cyst across the intervertebral foramen was difficult, because such cases were extremely rare, and the patient had no hydatid cysts of other organs and no previous history, the serological test was also negative. There were only abnormal MRI signals and significantly enhanced septum of paravertebral and intervertebral foramina. Therefore, it is easy to be misdiagnosed. We collected the detailed data of all published cases of primary extradural intraspinal together with paravertebral hydatid cyst in Table 1 (exclude primary involvement of other organs and vertebrae. Soft tissue involvement without bony origin and some literature did not mention whether it originated from other organs, which we viewed with skepticism), there were only 11 cases in all, but there were many early misdiagnosed cases among them. Therefore, the early correct diagnosis of this disease was much more important. There should be differential diagnosis of the disease from schwannoma and neurofibromatosis (10, 20): the lesion was not multiloculated, multiple loculations being more common with hydatid cyst. Hydatid cysts contain the hydatid fluid component and tend to invade anatomical cavities. Besides, it does not show contrast enhancement but exhibit a cerebrospinal fluid-like signal intensity on MR imaging. Differential diagnosis is also needed to avoid misdiagnosis with the aneurysmal bone cyst, giant cell tumor of bone, isolated bone cysts, arachnoid cysts, fibrocystic diseases, chondrosarcomas, and tuberculosis, etc. (11). Therefore, in the pastoral area, patients with progressive back pain and progressive paraplegia or other spinal cord compression symptoms should be suggestive of the occurrence of the primary disease. If the patient also has a history of hydatid disease in other organ systems, the occurrence of this disease can be highly suspected.

**Table 1:** Details of all published cases of primary extradural intraspinal together with paravertebral hydatid cyst.

**Author (published year)**	**Age (years)**	**Country**	**Gender**	**Location**	**Serology**	**Neurological status**	**Surgery outcome**
Ranganadham et al. (1990) (12)	35	India	Female	T4–T5	(–)	Progressive paraplegia	Improved
Tekkök et al. (1993) (13)	54	Turkey	Male	L2–L5	\	Cauda equina syndrome	No change
Hamdan et al. (2000) (14)	35	Iraq	Female	T7–T8	(–)	Paraparesis	Complete recovery
Awasthy and Chand (2005) (15)	15	India	Male	T4–T5	(–)	Paraparesis	Complete recovery
Gopal et al. (2007) (16)	38	India	Male	T2–T3	ELISA (–)	Paraparesis	Complete recovery
Joshi et al. (2014) (11)	28	Iraq	Male	T5–T6	\	Paraparesis	\
Assefa et al. (2014) (17)	16	Ethiopia	Male	\	\	Paraparesis	Improved
Karakasli et al. (2015) (6)	17	Turkey	Male	T3–T4	ELISA/Western blot (–)	Paraparesis	Complete recovery
Gennari et al. (2016) (18)	25	Romanian	Female	T8–T10	\	Paraparesis	Complete recovery
Antoniades et al. (2017) (19)	65	Greece	\	L2–L5	IHA (+)	Paraparesis	Improved/Incontinenza
Dighe et al. (2018) (10)	40	India	Female	T7–T8	\	Back pain	Improved
This case (2020)	42	China	Male	T12–L2	ELISA (–)	Back pain paraparesis	Complete recovery

*Exclude primary involvement of other organs and vertebrae. Soft tissue involvement without bony origin and some literature did not mention whether it originated from other organs, which we viewed with skepticism*.

The diagnosis of hydatid disease depends on radiology, biology, and serology examinations. Serological diagnosis is usually performed by Enzyme-Linked Immunosorbent Assay (ELISA), Western Blotting, Indirect Hemagglutination Assay (IHA), and Polymerase Chain Reaction (PCR). The sensitivity rates of serological diagnosis of the liver, lungs, and other organs are 80–100%, 50–56%, and 25–56%, respectively (6). Among the 11 cases reviewed, only 1 presented positive serological for IHA, while most of the other cases presented negative serological diagnosis and a few pieces of literature did not mention serological diagnosis. Because the serological test is less sensitive to echinococcosis of the nervous system, the characteristic imaging performance can also support the diagnosis, even if the serological test is negative. So, medical imaging is the main basis for the diagnosis of patients with a suggestive medical history (living in an endemic area) and clinical manifestations. CT or MRI examination is of great significance for the localization, qualitative, and quantitative diagnosis of this disease. Bone destruction has no specificity in imaging, it can be osteolytic destruction, vertebral deformities, or hyperplastic changes, and these changes are more prominent in the thoracic segment. And the symptoms may present in different nerve roots or spinal cord compressions depending on the occurrence of different segments of the spine. According to the statistics, the different symptom of spinal cord compression rate of paraparesis at presentation (61–73%), associated or not with back pain (27.8–43%), bladder dysfunction (11.1–32%), sensory loss (24%), and radicular pain (27–60%) (18). In our case, the patient's spinal cord compression symptoms showed lower back pain and paraparesis.

Surgical removal of the lesion is the best choice for the treatment of spinal hydatid disease, especially for the treatment of patients with spinal cord compression symptoms, surgical removal of the lesion is still the “gold standard.” For the operative approach selection, the lesion is usually deep in the side of the spinal bypass incision approach, which makes it difficult to quickly enter the lesion area and may cause retroperitoneal spread during the operation. Therefore, we need to choose the posterior straight incision approach of the affected spine segment, which can quickly enter the corresponding lesion area and facilitate the complete resection of the lesion. And it is also more convenient for spinal fusion and fixation. For the resection, the removal of hydatid cysts must be thorough, as much as possible to remove all endocyst in the field of surgery. During the operation, spinal cord injury must be avoided, and care should be given to prevent the puncturing of the endocyst wall. Finally, spinal fusion and fixation should be performed to restore spine stability while removing the lesion. Because the damage of hydatid to the spinal cord is mainly mechanical compression, rather than invasive damage. So, if it can be early detection and early surgical removal, the prognosis will be better.

In our case, the lesions occurred in the extradural, and the intraspinal lesions were small, with a clear boundary between the internal and external spinal canal and relatively limited adhesion around the surrounding tissues. So, the capsule can be completely removed. Remove the ectocyst completely and avoid residuum of the endocyst, then the disease could be cured. Besides, due to the slow onset of this patient, the large volume of the lower lumbar spinal canal, and it has a certain buffering effect, hydatid cysts extending along the nerve root sheath to the outside of the spine also play a certain role in decompression. And through the fusion fixation of the spine, the patient can do off-bed activity earlier. Therefore, the neurological function recovered well in this case after the operation.

Due to the limited space in the spinal canal, if the lesion in the spinal canal is large or at a high position, to avoid excessive compression of the spinal cord during intraoperative operation, the ectocyst can be cut and gauze can be used to protect the surrounding tissues, suction out the fluid of endocyst to decompression, and remove the ectocyst in a whole. If the endocyst ruptures during the operation, the rate of recurrence will be higher. Therefore, it is necessary to avoid intraoperative contamination as much as possible to prevent the hydatid cyst from rupturing and causing it to be planted in the surrounding tissues. Anti-hydatid drugs and appropriate hormones should be used as adjuvant therapy before and after surgery to avoid the occurrence of anaphylactic shock. After the removal of the lesions, it should be washed with hydrogen peroxide and 5% hypertonic saline to prevent recurrence or reduce the recurrence rate, then use dexamethasone water to avoid serious allergic reactions. All these measures are used to avoid various symptoms caused by allergic reactions or overflow of the content of the hydatid head section, such as itching, urticaria, edema, dyspnea, asthma, vomiting, diarrhea, colic abdominal pain, and even anaphylactic shock.

## Conclusion

Hydatid cyst across the intervertebral foramen has been reported only sporadically in the literature, and the primary cyst is extremely rare. For diagnosis, hydatid cyst disease should be considered when imaging findings indicate cystic changes in the spinal region and the patient has a pastoral life history, even if the serology test is negative. For treatment, the main method is still surgical treatment, and great attention should be paid to completely remove the cyst to avoid extravasation of the cystic fluid to cause spread.

As the incidence of this disease is extremely low, rarely encountered in clinical practice, so this case would be reported in the hope of improving the understanding, diagnose it and treat it in time.

## Data Availability Statement

The original contributions presented in the study are included in the article/supplementary material, further inquiries can be directed to the corresponding author/s.

## Ethics Statement

Written informed consent was obtained from the individual(s) for the publication of any potentially identifiable images or data included in this article.

## Author Contributions

YT and MJ performed the image data analyses and wrote the manuscript. XS contributed to perform the operation and give the manuscript preparation. YH helped perform the analysis with constructive discussions. LJ helped for giving the literature review. All authors contributed to the article and approved the submitted version.

## Conflict of Interest

The authors declare that the research was conducted in the absence of any commercial or financial relationships that could be construed as a potential conflict of interest.
